# Low Rice Intake Is Associated with Proteinuria in Participants of Korea National Health and Nutrition Examination Survey

**DOI:** 10.1371/journal.pone.0170198

**Published:** 2017-01-12

**Authors:** Se Jin Lee, So Young Lee, Su Ah Sung, Ho Jun Chin, Sung Woo Lee

**Affiliations:** 1 Department of Nephrology, Internal Medicine, Nowon Eulji Medical Center, Eulji University, Seoul, Korea; 2 Department of Internal Medicine, Seoul National University Bundang Hospital, Seongnam, Korea; 3 Department of Internal Medicine, Seoul National University Postgraduate School, Seoul, Korea; University of Colorado Denver School of Medicine, UNITED STATES

## Abstract

Little is known about the risk factors of proteinuria in the Asian population. On the basis of the association between rice intake patterns and chronic diseases, we hypothesized that rice intake patterns are associated with proteinuria in the Asian population. Data, including data regarding rice intake frequency and dipstick urinalysis results, from the Korea National Health and Nutrition Examination Survey in 1998, 2001, 2005, and 2007 were analyzed. The study involved 19,824 participants who were older than 20 years of age. Low rice intake was defined as consumption of rice ≤ 1 time/day. Proteinuria was defined as dipstick urinalysis protein ≥ 1 positive. Among the 19,824 participants, the prevalence of low rice intake and proteinuria were 17.3% and 2.9%, respectively. The low rice intake group showed a higher rate of proteinuria than the non-low rice intake group did (3.8% vs. 2.7%, *P* < 0.001). In multivariate logistic regression analysis, the odds ratio (OR) of low rice intake for proteinuria was 1.54 (95% confidence interval (CI): 1.25–1.89; *P* < 0.001). Low rice intake was also independently associated with high blood pressure (OR: 1.43, 95% CI: 1.31–1.56; *P* < 0.001) and diabetes (OR: 1.43, 95% CI: 1.27–1.62; *P* < 0.001). In conclusion, low rice intake was found to be independently associated with proteinuria in the Asian population, which might have been affected by the associations of low rice intake with high blood pressure and diabetes. Future prospective studies are needed to confirm the results of this study.

## Introduction

Proteinuria is a marker of kidney damage [1]. Since measuring proteinuria quantitatively in a large population is not always possible, many studies have measured proteinuria semi-quantitatively by using dipstick urinalysis. Proteinuria measured using dipstick urinalysis is also a well-known predictor of future end-stage renal disease (ESRD) [2, 3] and all-cause mortalities [4–6], including those due to cardiovascular diseases. Although proteinuria can be reduced by the inhibition of the renin-angiotensin-aldosterone system (RAAS) [7], administration of RAAS inhibitor to people with proteinuria, who are otherwise healthy, is not always possible. It has been debated [8] that Asians may be at an increased risk of proteinuria than people of other ethnicities [9, 10]. Therefore, it is important to identify modifiable risk factors for proteinuria, particularly in Asians.

Rice is a staple food in many Asian countries. However, the consumption of rice has decreased owing to rapid Westernization [11, 12]. High rice intake is associated with decreased risk of hypertension [13–15]. It has been debated [16] that frequent rice intake can be associated with a decreased risk of diabetes [17, 18]. Since hypertension and diabetes are important causes of proteinuria [8, 19, 20], we hypothesized that low rice intake was associated with proteinuria, as measured using dipstick urinalysis. To test our hypothesis, this study was performed using data from Korea National Health and Nutrition Examination Survey (KNHANES), a nationwide and government-administered survey.

## Materials and Methods

### Study population

The KNHANES is a cross-sectional and nationally representative survey on the health and nutritional status of the non-institutionalized Korean civilian population. The study protocol complied with the Declaration of Helsinki. Full approval of the study was obtained from the Institutional Review Board of the Statistics Korea in KNHANES 1998–2005 (IRB number: 11702) and the Korea Centers for Disease Control & Prevention in KNHANES 2007 (IRB number: 2007-02CON-04-P). The protocol comprised a health-questionnaire survey, health examination, and nutrition survey. We used the data from the 1998, 2001, 2005, and 2007 KNHANES. The KNHANES in 1998, 2001, and 2005 were performed for 10 weeks, whereas the 2007 survey was performed year-round. With the change of the data collecting system in June 2007, the survey in 2007 only began in July 2007, with only half of the desired results obtained in 2007. Among 34,383 participants who completed the nutrition survey, 19,824 (57.7%) were 20 years or older and were not missing data regarding rice intake frequency and dipstick urinalysis result were included in the analysis: 7,250 (62.3%) in 1998; 5,492 (55.2%) in 2001; 4,592 (52.3%) in 2005; and 2,490 (62.1%) in 2007.

### Food intake assessment

Food intake was assessed using a Food Frequency Questionnaire (FFQ). The frequency of consumption was assigned into the following 10 categories: 1, 2, or 3 times per day, 4–6 times per week, 2–3 times per week, once per week, 2–3 times per month, once per month, or 6–11 times per year, without any relationship with food items. The survey did not consider the quantity of rice consumed, such as portion size, and the results were non-quantitative. In KNHANES, the surrogate measurement for food intake was single-day 24 h recall (24H-RC). In addition to its inherent limitations, such as poor reflection of day-to-day variation [21], the 24H-RC information was collected differently between 1998–2001 and 2005–2007, which might have caused a considerable bias. Therefore, we decided not to use the 24H-RC results in this study. Instead, we used only the FFQ results to define food intake. Although FFQ is non-quantitative, it is a good representative of usual food intake [21].

### Measurements and definitions

Urine protein was measured via dipstick urinalysis by using a multistix reagent strip (YD Diagnostics corp., Korea) and analyzed using a Urisacn auto-analyzer (YD Diagnostics corp., Korea). Results were reported on a semi-quantitative scale from negative to 4+. Serum glucose and creatinine levels were measured using a Hitachi-747 autoanalyzer (Hitachi, Japan) in 1998 and 2001 and an ADIVIA 1650 analyzer (Siemens, U.S.) in 2005 and 2007. Blood pressure (BP) was measured using the standard protocol in accordance with the recommendation [22, 23]. Two readings were obtained and their mean value was reported as the final BP. Body mass index (BMI) was calculated as (weight in kg)/(height in m)^2^. Estimated glomerular filtration rate (eGFR) was calculated using an equation of the Chronic Kidney Disease Epidemiology Collaboration [24].

Proteinuria was defined when the result of dipstick urinalysis test was ≥ 1+. High BP was defined as a systolic BP ≥ 130 mmHg or a diastolic BP ≥ 80 mmHg. Diabetes was defined as fasting glucose level ≥ 126 mg/dl or administration of oral anti-diabetic drugs or insulin at the time of interview or a physician’s diagnosis of diabetes. Obesity was defined as a BMI ≥ 25 kg/m^2^. Renal hyperfiltration was defined as the highest quartile of eGFR in the whole population [25]. High income was defined as above the highest quartile in each survey year. Low rice intake was defined as consumption of rice ≤1 time per day. The intakes of fruits, vegetables, meat, fish, and snacks were defined as consumption of these foods ≥ 1 time per day.

### Statistical analysis

Data are presented as the means ± standard deviation for continuous variables and percentages for categorical variables. Differences were analyzed using a Chi-square test for categorical variables and Student’s t-test for continuous variables. *P* < 0.05 was considered statistically significant. Variables used in the multivariate logistic regression analysis for proteinuria were those found to be statistically significant in univariate logistic regression analysis. Pearson correlation test was performed to determine the potential collinearity among selected variables. All analyses and calculations were performed using SPSS Statistics (version 22; IBM, USA).

## Results

Among the 19,824 participants, the prevalence of low rice intake and proteinuria were 17.3% and 2.9%, respectively. Baseline characteristics of the study population according to rice intake are summarized in Table 1. Younger age and lower participation by males was observed in the low rice intake group than in the non-low rice intake group. More people in the low rice intake group had high BP, diabetes, and renal hyperfiltration than in the non-low rice intake group. People in the low rice intake group were more likely to live in urban areas. They had lower incomes than those in the non-low rice intake group. People with low rice intake had meals more frequently with a more westernized food pattern. They ate more meat and snacks, but less fruits or vegetables, than did those with non-low rice consumption.

**Table 1:** Baseline characteristics of study population by rice intake.

Variables (n = 19824)	Non-low rice intake (n = 16,392)	Low Rice intake (n = 3,432)	*P*
Age (years)	46.7 ± 15.7	45.2 ± 15.4	<0.001
Male sex (%)	44.3	40.1	<0.001
Survey year (1998–2001, %)	58.0	94.1	<0.001
**Socioeconomic status**			
Urban region (%)	41.4	46.4	<0.001
College graduate (%, n = 19,782)	25.9	24.3	0.052
High income (%, n = 19,375)	21.0	12.4	<0.001
**Comorbidity**			
High blood pressure (%, n = 19,341)	35.6	40.0	<0.001
Anti-hypertensive drug use (%, n = 19,716)	10.1	6.7	<0.001
Diabetes (%, n = 19,594)	9.3	11.6	<0.001
Obesity (%, n = 19,775)	30.0	28.7	0.124
Proteinuria (%)	2.7	3.8	<0.001
Renal hyperfiltration (%, n = 19,681)	18.1	28.2	<0.001
eGFR (ml/min/1.73m^2^, n = 19,681)	83.9 ± 16.1	88.8 ± 16.2	<0.001
**Food intake pattern**			
Meal 3 times a day (%, n = 19,804)	72.8	74.5	0.037
Fruits or vegetable intake (%, n = 19,816)	93.8	92.1	<0.001
Meat intake (%, n = 19,882)	3.2	4.3	0.001
Fish intake (%, n = 19,819)	18.9	19.8	0.259
Snacks intake (%, n = 19,802)	69.1	73.1	<0.001

eGFR: estimated glomerular filtration rate.

Values are presented as the mean ± standard deviation for continuous variables and % for categorical variables. Differences were evaluated via t-test for continuous variables and chi-square test for categorical variables. The Total numbers of participants for each variable is expressed in parenthesis. The missing rate was less than 2.5% for all variables.

To determine the factors associated with proteinuria, we performed logistic regression analysis (Table 2). On the basis of this analysis, low rice intake was found to be a significant risk factor for proteinuria in multivariate analyses after adjusting for factors, such as age, sex, high BP, antihypertensive drug use, diabetes, obesity, renal hyperfiltration, fruit or vegetable intake, and college graduation [odds ratio (OR): 1.54, 95% confidence interval (95% CI): 1.25–1.89; *P* < 0.001].

**Table 2:** Factors associated with proteinuria.

	Univariate		Multivariate	
	OR (95% CI)	*P*	OR (95% CI)	*P*
Age (per 10 years increase)	1.15 (1.09–1.21)	<0.001	0.92 (0.86–0.99)	0.033
Sex (male vs. female)	1.20 (1.01–1.41)	0.034	1.20 (1.00–1.44)	0.045
High BP (yes vs. no)	1.98 (1.67–2.34)	<0.001	1.42 (1.17–1.73)	<0.001
Antihypertensive drug use (yes vs. no)	3.09 (2.53–3.78)	<0.001	2.31 (1.81–2.94)	<0.001
Diabetes (yes vs. no)	2.70 (2.19–3.32)	<0.001	1.98 (1.58–2.48)	<0.001
Obesity (yes vs. no)	1.64 (1.39–1.95)	<0.001	1.32 (1.10–1.59)	0.003
Renal hyperfiltration (yes vs. no)	0.72 (0.57–0.91)	0.005	0.78 (0.60–1.02)	0.070
Meal 3 times a day (yes vs. no)	1.04 (0.86–1.26)	0.671	-	-
Rice intake group (low vs. non-low)	1.46 (1.20–1.78)	<0.001	1.54 (1.25–1.89)	<0.001
Fruits or Vegetables intake (yes vs. no)	0.71 (0.53–0.96)	0.024	0.75 (0.55–1.03)	0.074
Meat intake (yes vs. no)	0.99 (0.62–1.57)	0.955	-	-
Snack intake (yes vs. no)	1.01 (0.84–1.21)	0.916	-	-
Urban region (yes vs. no)	0.97 (0.82–1.15)	0.745	-	-
College graduate (yes vs. no)	0.64 (0.52–0.80)	<0.001	0.72 (0.56–0.92)	0.008
High income (yes vs. no)	0.87 (0.69–1.08)	0.202	-	-
Survey year (2005–2007 vs. 1998–2001)	0.91 (0.76–1.09)	0.303	-	-

OR: odds ratio; CI: confidence interval; BP: blood pressure.

Variables with *P* < 0.05 in univariate analysis were included in the multivariate analysis.

In the analysis of the effect of low rice intake on high BP and diabetes, low rice intake was also found to be significantly associated with increased odds for high BP and diabetes, with ORs (95% CI) of 1.43 (1.31–1.56, *P* < 0.001) and 1.43 (1.27–1.62, *P* < 0.001), respectively (Table 3).

**Table 3:** Odds ratio of high blood pressure and diabetes in low rice intake group.

	High blood pressure*		Diabetes*	
	Adjusted OR (95% CI)	*P*	Adjusted OR (95% CI)	*P*
Rice intake (low vs. non-low)†	1.43 (1.31–1.56)	<0.001	1.43 (1.27–1.62)	<0.001
Age (per 10 years increase)†	1.64 (1.60–1.68)	<0.001	1.49 (1.43–1.55)	<0.001
Sex (male vs. female)†	2.23 (2.08–2.39)	<0.001	1.49 (1.35–1.65)	<0.001
High blood pressure (yes vs. no)†	-	-	1.39 (1.25–1.55)	<0.001
Anti-hypertensive drug (yes vs. no)†	3.08 (2.72–3.49)	<0.001	1.77 (1.55–2.03)	<0.001
Diabetes (yes vs. no)†	1.33 (1.19–1.49)	<0.001	-	-
Obesity (yes vs. no)†	2.11 (1.97–2.27)	<0.001	1.74 (1.57–1.93)	<0.001
Renal hyperfiltration (yes vs. no)†	1.21 (1.10–1.34)	<0.001	1.27 (1.07–1.50)	0.006
Proteinuria (yes vs. no)†	1.41 (1.16–1.73)	0.001	1.96 (1.56–2.47)	<0.001

OR: odds ratio; CI: confidence interval.

The above major comorbidities associated with either high blood pressure or diabetes in univariate logistic regression analysis were entered as covariates in multivariate logistic regression analysis.

*effects of the analysis,

^†^ causes of the analysis.

In subgroup analysis that controlled for factors, such as age, sex, high BP, diabetes, and obesity, the effect of low rice intake on proteinuria was only evident in people with age ≥ 65 years, people who are women, and people without diabetes. Rice intake was consistently associated with proteinuria regardless of the status of high BP or obesity (Table 4).

**Table 4:** Subgroup analysis of the association between low rice intake and proteinuria.

Subgroups		Adjusted OR (95% CI)*	*P*
Age	≥65 years (n = 16,632)	1.55 (1.24–1.95)	<0.001
	<65 years (n = 3,192)	1.48 (0.92–2.40)	0.106
Sex	Men (n = 8,635)	1.33 (0.96–1.83)	0.083
	Women (n = 11,189)	1.73 (1.32–2.27)	<0.001
High blood pressure	No (n = 12,310)	1.65 (1.23–2.22)	0.001
	Yes (n = 7,301)	1.45 (1.09–1.92)	0.012
Diabetes	No (n = 17,691)	1.66 (1.32–2.09)	<0.001
	Yes (n = 1,903)	1.14 (0.71–1.82)	0.597
Obesity	No (n = 13,878)	1.58 (1.22–2.05)	0.001
	Yes (n = 5,897)	1.45 (1.04–2.03)	0.029

OR: odds ratio; CI: confidence interval.

*OR of low rice to non-low rice intake group for proteinuria was calculated via multivariate logistic regression analysis after adjusting for age, sex, high BP, anti-hypertensive drug use, diabetes, renal hyperfiltration, fruits or vegetables and college graduate.

## Discussion

Asian people might have an increased risk for proteinuria. There are several effective medications to lower the rate of proteinuria. However, medication is not always useful. In this context, we identified modifiable risk factors of proteinuria, particularly in the Asian population. It has been debated that high rice intake might be associated with decreased risk of chronic diseases such as hypertension and diabetes. Therefore, we hypothesized that low rice intake was associated with proteinuria. To test our hypothesis, we performed this study by using the data from KNHANES and found that low rice intake was significantly associated with proteinuria. Elder people, women, and people without diabetes might be more sensitive in a subgroup for the association between rice intake and proteinuria. To the best of our knowledge, this is the first study that reports such an association.

The prevalence of proteinuria was 1.4 times higher in people with low rice intake than in those with non-low rice intake. In multivariate analysis after adjusting for covariates, the OR of low rice intake for proteinuria was 1.54, which was quite similar to other known risk factors, such as high BP (OR = 1.42), diabetes (OR = 1.98), and obesity (OR = 1.32). On the basis of this result, we postulated that the potential risk associated with low rice intake for proteinuria may not only be statistically significant, but also be clinically significant. The results of this study are in agreement with the results of the study by Kempner et al., thereby suggesting that a rice-rich diet could be used to treat kidney disease [26]. However, compared to our study, their study was focused on filtration capacity instead of proteinuria. In addition, their study design was out of date.

Currently, the reason why low rice intake is associated with proteinuria remains unclear. We observed that low rice intake is associated with increased odds of high BP and diabetes, which is similar to the results of previous studies. Ever since the 1940s, a rice-rich diet is thought to be associated with decreased risk of hypertension [14, 27–29]. In a recent study, Shi et al. examined the association between rice intake and the risk of metabolic syndrome development in Chinese adults [13]. They found that the ORs for high BP in people with rice intake 201–400 g/day and ≥ 401 g/day compared to those with rice 0–200 g/day were 0.68 and 0.58, respectively, after adjusting for covariates [13]. Son et al. suggested that reducing the amount of rice intake per meal could prevent postprandial blood pressure decreases in the older Korean population [30]. Although debatable [16], increased rice intake might be associated with a lower risk of diabetes. Soriguer et al. recently suggested that subjects who eat rice more frequently have a lower risk of developing diabetes 6 years later (OR: 0.43, *P* = 0.04) in a Spanish population-based cohort [18]. Dong et al. have found an inverse association between the highest (versus lowest) tertile of rice intake and diabetes in Central China (OR: 0.59, *P*-trend = 0.03) [17]. Since high BP and diabetes were significantly associated with proteinuria in our study and in previous studies [8, 19, 20], the clear association of low rice intake with high BP and diabetes might be a good explanation for the relationship between low rice intake and proteinuria.

Socioeconomic status (SES) may be another factor that explains the significant association between rice intake and other dietary behaviors. In the current study, people with low rice intake tended to have more westernized diet than those with non-low rice. They ate more meat and snacks, but less fruits or vegetables. Diets that are rich in fruits and vegetables might be able to reduce proteinuria directly. Jacobs Jr et al. have studied the effect of fruit and vegetable diet on urinary albumin excretion rate (AER) [31]. After eight weeks of intervention, the decrease in AER occurred only in those with a fruit and vegetable diet, in a pattern that was distinct from the decrease in BP. Generally, low SES is associated with poor health outcomes [32, 33]. In this study, college graduates were associated with decreased odds of proteinuria. Since people with a low rice intake tended to have low SES (i.e., low levels of income and education), SES might affect the association between rice intake and proteinuria.

Our study has several limitations. First, proteinuria, the main study outcome, was defined using urine dipstick results, a semi-quantitative proteinuria estimation whose sensitivity and specificity have been thought to be insufficient [34]. Although the reliability issue of the dipstick technique affects the study results, we postulate that the effect might be minimal since the sensitivity and specificity of the dipstick technique varies in different clinical settings. In some studies, the sensitivity and specificity of the dipstick urinalysis protein ≥ 1+ in detecting the albumin to creatinine ratio ≥ 300 has improved to ≥ 90% [35, 36]. Moreover, proteinuria measured using the dipstick technique itself is a well-validated risk factor for ESRD [2, 3] and mortality [4–6], and is still being used as the main factor or outcome [37–40]. Therefore, this limitation might be marginal. Second, the definition of rice intake was based on non-quantitative FFQ. Its frequency does not always reflect the amount. Although it is non-quantitative, FFQ is a good way to represent the usual intake of food [21]. The definition of rice consumption using FFQ has been used in previous studies [18]. Third, the cross-sectional design of our study may limit causal relationships. Therefore, our results need to be carefully interpreted. We believe that the hypothesis generated by our study must be confirmed in future prospective studies. Finally, the single ethnicity in our study might limit the generalizability of our study results.

In conclusion, low rice intake was found to be independently associated with proteinuria in Asian adults. The significant association between low rice intake and chronic diseases, particularly high BP and diabetes, might account for such a relationship between low rice intake and proteinuria. Though rice is a staple food in many Asian countries, its consumption has decreased recently. Therefore, increasing rice intake might have a beneficial effect on the treatment of proteinuria, particularly in elderly people, women, and people without diabetes, in the Asian population. Further studies are needed to confirm these findings.

## Supporting Information

S1 Dataset Dataset of the current study. (XLSX)
